# Protocol for the Implementation of a Targeted Maternal and Newborn Service Delivery Bundle in Sierra Leone

**DOI:** 10.3390/mps9030096

**Published:** 2026-06-10

**Authors:** Robert B. Clark, Joseph Odu, Annette Ofodum, Rondi Anderson

**Affiliations:** 1 Department of Public Health, Brigham Young University, Provo, UT 84602, USA; 2 Africa Regional Office, Project HOPE, Washington, DC 20036, USA; jodu@projecthope.org; 3 Sierra Leone Country Office, Project HOPE, Freetown, Sierra Leone; aofodum@projecthope.org; 4 Reproductive Health, Project HOPE, Washington, DC 20036, USA; randerson@projecthope.org

**Keywords:** neonatal mortality, postpartum hemorrhage, implementation science, bubble CPAP, Sierra Leone, essential newborn care, obstetric ultrasound

## Abstract

Sierra Leone faces persistently high neonatal and maternal mortality rates, driven largely by delayed recognition and treatment of newborn respiratory distress and postpartum hemorrhage. In this protocol, we describe the planned implementation of a bundle of maternal and newborn clinical practices over a 36-month period across nine public health facilities in the Greater Freetown area and Bo District to address these critical gaps. The service delivery improvements include the World Health Organization (WHO) Essential Newborn Care Course (ENCC) Parts 1 and 2; Vayu bubble continuous positive airway pressure (bCPAP) and oxygen blenders for respiratory support; the WHO Postpartum Hemorrhage package; and obstetric risk stratification using point-of-care ultrasound (POCUS) and complementary diagnostics for maternal care improvement. We anticipate that this bundle of evidence-based clinical tools and training, reinforced by mentorship, structured checklists, and low-dose high-frequency (LDHF) practice, will significantly reduce perinatal and maternal mortality and morbidity. The bundle will be evaluated using a Hybrid Type 1 effectiveness-implementation design, utilizing routine health information system data, supplemented by project registers, skills assessments, and observations. By aligning with the Ministry of Health’s Child Survival Action Plan, the aim of this project protocol is to provide a sustainable and scalable model for reducing preventable maternal and newborn deaths in resource-constrained settings.

## 1. Introduction

### 1.1. Background

Neonatal mortality (infant death within 28 days of birth) remains a significant health concern worldwide, accounting for nearly half of all deaths in children under five years of age, with the majority occurring in low-to-middle-income countries (LMICs) [1,2]. In Sierra Leone, the neonatal mortality rate (NMR) remains high at 31 per 1000 live births, far exceeding the Sustainable Development Goal 3.2 (SDG) target of 12 per 1000 [3,4]. Reducing neonatal mortality and morbidity is thus a high priority.

Neonatal respiratory distress is the leading cause of neonatal morbidity and mortality in the first week of life. Immediate recognition of respiratory distress, followed by prompt resuscitation and follow-on care, is critical for survival [5,6,7,8,9]. The results of previous studies have demonstrated that implementation of bundled newborn care interventions, including resuscitation training and respiratory support, can significantly reduce neonatal mortality. For example, the “Safer Births Bundle of Care” program in Tanzania, which implemented low-dose, high-frequency resuscitation training and skills mentoring for midwives, recently demonstrated a 40% reduction in neonatal deaths in the first week of life [10].

Continuous positive airway pressure (CPAP), delivered as either mechanical or bubble CPAP, plus oxygen blenders improve neonatal respiratory distress outcomes by preventing hyperoxic lung injury and retinopathy of prematurity. In a separate study from Tanzania, Vayu brand bubble CPAP (bCPAP) implementation was associated with immediate improvement in respiratory function [11]. While these and similar findings validate the hypothesis that bundling evidence-based clinical tools with skills mentoring and practice can drive rapid improvements in survival in high-burden settings, the implementation of advanced newborn care in Sierra Leone remains limited due to equipment shortages and gaps in provider training [12,13,14,15,16]. In light of the country’s position among the 15 countries with the highest NMRs worldwide, overcoming these barriers is an urgent priority.

Meanwhile, the maternal mortality rate (MMR) in Sierra Leone remains unacceptably high at 443 per 100,000 live births, placing it among the worst 15 countries worldwide [2,3,17]. Postpartum hemorrhage (PPH) is the leading global cause of maternal mortality and is particularly deadly in resource-constrained settings, where detection and treatment can be delayed [3].

Intrapartum complications, such as hemorrhage, labor dystocia, fever, abnormal lie, and fetal distress, may lead to perinatal asphyxia, worsening outcomes for both the mother and baby [18,19,20,21,22]. To mitigate these risks, the integration of obstetric ultrasound in LMIC maternity units has proven highly valuable [23]. Evidence indicates that routine ultrasound use significantly improves diagnostic accuracy and risk stratification for high-risk conditions, such as placenta previa, malpresentation, and multiple gestations, enabling timely referrals and appropriate clinical management before emergencies arise [23]. Thus, more effective recognition and treatment of hemorrhage, combined with enhanced maternal risk assessment and case management, can improve maternal outcomes.

**Figure 1:**
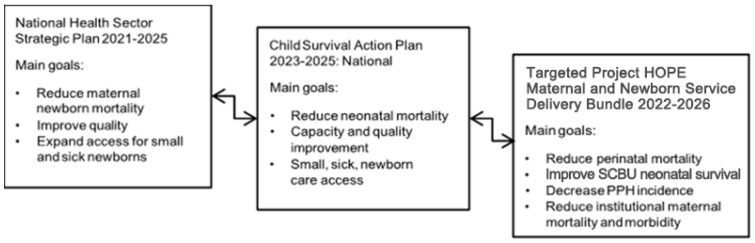
Project alignment with national goals.

The aim of this protocol is to describe a targeted newborn and maternal bundle that will be implemented in Sierra Leone, as shown in Figure 1. The newborn bundle combines improving and sustaining high-quality neonatal resuscitation with utilization of bCPAP for respiratory distress treatment. The maternal bundle combines improved intrapartum risk assessment with high-quality prevention and treatment of maternal hemorrhage.

### 1.2. Strategic Alignment and Policy Context

The bundle operationalizes two national frameworks, including the Sierra Leone National Health Sector Strategic Plan 2021–2025 and the Ministry of Health and Sanitation (MoHS) Child Survival Action Plan 2023–2025, both of which prioritize reductions in under-five and maternal mortality through strengthened newborn services [24,25]. The MoHS has established special care baby units in 16 districts and scaled up Helping Babies Breathe nationally [26]. Two implementation gaps remain. First, the more comprehensive WHO Essential Newborn Care Course (ENCC), particularly Part 2, covering the first 24 h of life has not been introduced [27]. Second, the WHO Postpartum Hemorrhage Package has not been operationalized as an integrated bundle linking blood-loss quantification, intrauterine balloon tamponade, and active management of the third stage of labor. This protocol addresses both gaps; Figure 1 maps each bundle component to its corresponding national policy objective.

The maternal component aligns with both the national plan and global best practices by implementing the WHO Postpartum Hemorrhage Package and transitioning care from simple management to a comprehensive PPH “bundle” approach. Innovative maternal triage and risk assessment strategies address potential upstream sources of intrapartum complications. Aligned with these national priorities, the primary objective of implementing this targeted bundle is to improve maternal and newborn outcomes. Figure 1 shows the alignment of the maternal and newborn bundle with national goals.

### 1.3. Implementation Logic Model

The implementation logic follows a pathway in which resource inputs lead to enhanced clinical capacity and improved outcomes.

- Inputs: Newborn: Vayu bCPAP, oxygen blenders, resuscitation equipment, monitors, oxygen concentrators, and consumables. Maternal: hemorrhage quantification and treatment tools, Butterfly ultrasound, Hemocue, heart tone Dopplers, and consumables.
- Activities: Training in ENCC Parts 1 and 2, Vayu bCPAP training, WHO PPH Course, and basic ultrasound diagnosis training; establishment of facility-based “Champions” and LDHF practice corners. Monthly mentoring, supportive supervision, and scale-up assistance.
- Outputs: We anticipate timely resuscitation (within one minute) and improved treatment of respiratory distress, in addition to improved maternal risk stratification and management of PPH.
- Outcomes: We anticipate a reduction in perinatal mortality, improved SCBU neonatal survival, and decreases in PPH incidence, institutional maternal mortality, and morbidity.
- Impact: We anticipate a sustained reduction in maternal and neonatal mortality and morbidity, contributing to SDG targets.

This implementation logic model is based on the following assumptions:

- There will be sustained political support for this project.
- Supply chain systems will ensure maternal and newborn commodity security across supported facilities.
- Staff will remain committed to project implementation.

**Figure 2:**
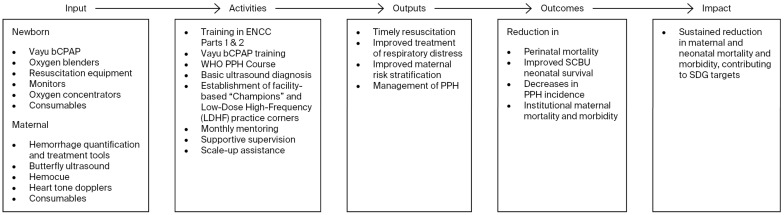
Implementation logic model.

The main components of the project plan, as outlined above, are shown in Figure 2.

### 1.4. Objectives

The primary objective of implementing a targeted service delivery bundle is to enhance maternal and newborn outcomes in a cohort of nine hospitals over a three-year period. These service delivery improvements aim to achieve the following:

- Reduce neonatal mortality: Achieve a 50% reduction in neonatal deaths within the first 24 h and a 10% reduction in intrapartum or “fresh” stillbirths compared to a baseline assessment.
- Reduce neonatal morbidity: Achieve a 25% reduction in neonatal transfers to advanced care.
- Improve neonatal clinical practice: Ensure that 80% of SCBU admissions with respiratory distress receive appropriate oxygen therapy.
- Reduce maternal morbidity: Achieve a 40% reduction in the incidence of postpartum hemorrhage and a 50% reduction in institutional maternal mortality.
- Improve obstetric clinical practice: Ensure that 90% of deliveries utilize active management of the third stage of labor with uterotonics to prevent hemorrhage.
- Strengthening diagnostic capacity: Deploy handheld ultrasound and point-of-care hemoglobin testing to improve obstetric risk stratification.
- Maintain competency: Implement a “train-the-trainer” model, mentoring, low-dose high-frequency practice, and supportive supervision to maintain high performance on knowledge and skills checks at 12-month intervals.

## 2. Materials and Methods

### 2.1. Implementation Design and Timeline

The longitudinal review of this protocol will utilize a Hybrid Type 1 effectiveness-implementation design. This approach allows for the primary review of the clinical effectiveness of the maternal and newborn bundle on patient outcomes, while simultaneously observing and gathering data on its feasibility, acceptability, and sustainability within the targeted facilities. The review employs a prospective, quasi-experimental before-and-after design.

Routinely collected service-delivery data from the three months immediately before implementation will be compared with data from the final three months of the 36-month implementation period, with continuous monitoring throughout. This design was selected for three reasons. First, the bundle is delivered as a facility-level package of training, equipment, mentoring, and supervision; patient-level randomization would not preserve intervention integrity. Second, withholding the bundle from comparable facilities is ethically difficult given Sierra Leone’s mortality burden and the established effectiveness of each component. Third, all participating facilities report to the national District Health Information System 2 (DHIS2), providing a long retrospective time-series that strengthens within-facility comparisons. Limitations arising from the absence of concurrent control are discussed in Section 3.5. Implementation will be divided into three phases:

- Preparatory Phase: Baseline analysis and equipment procurement. Based on these findings, a causal/bottleneck analysis of the adequacy of perinatal care quality will be conducted and shared with hospital administrations, and mitigation plans will be prepared.
- Implementation Phase (months 1–18): Training of trainers and equipment deployment. Training scale-up, mentoring, and supportive supervision, to include midwives, nurses, surgical and community health officers, and physicians (2024–2025).
- Sustainability Phase (months 19–36): Continue supportive supervision and transition to MoHS ownership (2026–2027).

### 2.2. Setting and Geographic Clusters

The bundle implementation will be conducted in nine public health facilities in Sierra Leone: eight in the Greater Freetown area and one in the city of Bo, including one tertiary hospital, four secondary hospitals, and four community health centers (CHCs), as described below and in Table 1.

Participating facilities:

- Tertiary: Princess Christian Maternity Hospital (PCMH)—Freetown.
- Secondary: Bo Government Hospital, Rokupa Government Hospital, King Harman Road Government Hospital, and Waterloo CHC (functioning as a secondary hospital).
- Primary (CHCs): Jenner Wright, Kissy, Ross Road, St. Anthony’s (Note: These CHCs do not perform cesarean sections or care for small/sick newborns.).

### 2.3. Implementation (The Service Delivery Bundle)

The service delivery bundle comprises a newborn package and a maternal package, supported by cross-cutting implementation strategies.

1. Newborn Care Package
  - Curriculum: Implementation of the World Health Organization Essential Newborn Care Course (ENCC) Parts 1 and 2.
  - Respiratory Support: Training and deployment of Vayu bCPAP systems and Vayu oxygen blenders to prevent hyperoxic lung injury and retinopathy of prematurity.
  - Equipment: Provision of bag-and-mask devices, suction units, heart rate monitors, and pulse oximeters, in addition to Vayu bCPAP systems, oxygen blenders, and consumables.
  - Kangaroo Mother Care (KMC): Expansion of KMC, to ensure neonatal euthermia, for the immediate care of small and sick newborns.
2. Maternal Care Package
  - PPH Management: Implementation of the WHO Postpartum Hemorrhage Package, including blood-loss quantification aids, intrauterine balloons, and anti-shock garments.
  - Advanced Diagnostic Equipment: Training and deployment of Butterfly iQ3 handheld ultrasound probes (POCUS) for delivery wards and point-of-care hemoglobin testing.
  - Risk Stratification: Enhanced triage and risk assessment using handheld ultrasound, improved use of fetal Dopplers and sphygmomanometers, and hemoglobin testing.
3. Cross-cutting Strategies
  - Respectful Care: Principles and practices of respectful care will be integrated into clinical training and mentoring activities.
  - Infection Control and Prevention (IPC) Supplies: Refresher training and supplies to improve water, sanitation, and hygiene practices.
  - Service Optimization: Facility-based teams will identify unique challenges and implement and document improved practices.
4. Implementation Strategy
  - Mentorship: Project HOPE will supply external mentors to cascade training and provide supportive supervision while observing patient care practices.
  - Champions: Two internal “Champions” will be recruited at each facility to support training scale-up and practice.
  - Practice Corners: Establishment of LDHF practice stations for skills retention.
5. Ancillary Services, Equipment, and Supplies
  - Data: Mentoring healthcare staff with documentation challenges and collecting and cleaning data.
  - Equipment: Ancillary equipment includes Dopplers, stethoscopes, pulse oximeters, sphygmomanometers, thermometers, glucometers, oxygen concentrators, heart rate monitors, resuscitation equipment, and training simulators.
  - Supplies: Consumables for bCPAP, hemoglobin, and urine testing, WASH equipment and supplies, personal protective equipment and sanitation supplies, hemorrhage kits and commodities, and printed materials.

### 2.4. Study Outcomes

To align with the Hybrid Type 1 effectiveness-implementation framework, the review metrics are strictly delineated into clinical effectiveness outcomes (measuring the health impact on mothers and newborns) and implementation outcomes (measuring the operational success, fidelity, and sustainability of the service delivery bundle).

#### 2.4.1. Clinical Effectiveness Outcomes

The primary clinical outcomes focus on immediate survival and severe morbidity:

- Primary Neonatal: Reduction in the neonatal mortality rate within the first 24 h.
- Primary Maternal: Reduction in the incidence of postpartum hemorrhage (≥500 mL).
- Secondary Outcomes: Fresh stillbirth rate, institutional maternal case fatality rate, neonatal transfer rate for advanced care, and survival rate of sick newborns admitted to the SCBU.

#### 2.4.2. Implementation Outcomes

Implementation success will be evaluated through metrics of fidelity, uptake, and sustainability:

- Fidelity and Adherence: The proportion of eligible SCBU admissions successfully initiated on Vayu bCPAP; the proportion of deliveries utilizing active management of the third stage of labor (AMTSL) with uterotonics; and the proportion of obstetric admissions receiving triage ultrasound scans.
- Uptake and Penetration: The total number of healthcare workers (midwives, nurses, physicians) successfully completing the WHO ENCC and PPH training modules against facility targets.
- Sustainability and Retention: The frequency of documented low-dose high-frequency (LDHF) practice sessions per facility; and objective skills retention measured via Objective Structured Clinical Examinations (OSCEs) at 12-month intervals.

### 2.5. Outcome Measures

Outcome indicators, definitions, and data sources are summarized in Table 2. Primary outcomes are institutional maternal mortality, neonatal mortality within 24 h of birth, intrapartum stillbirth, and PPH incidence. Secondary outcomes include process indicators (e.g., uterotonic coverage for AMTSL, proportion of obstetric admissions receiving triage ultrasound) and intermediate clinical indicators (e.g., proportion of sick newborns initiated on bCPAP, SCBU survival).

### 2.6. Data Collection

Data collection is stratified by outcome type:

- Clinical Data (See Table 2): Quantitative will be collected from existing Ministry of Health (DHIS2) systems and project-specific sources. Project data collectors will collaborate with medical record officers to extract granular clinical data from consecutive maternity and newborn registers across all nine facilities. A preliminary review for validation and missing entries will be followed by cross-verification. Outcomes observed during the implementation and sustainability periods will be compared with retrospective baseline data collected three months prior to training.
- Implementation and Educational Data (See Table 3): Quantitative implementation metrics, including training logs and low-dose high-frequency (LDHF) practice frequencies, will be extracted from facility-level project registers. Educational indicators, such as performance on objective structured clinical examinations (OSCEs) and skills retention at 12 months, will be collected and tabulated by the mentorship teams.
- Qualitative data: Qualitative insights regarding implementation barriers and skill retention will be obtained from selected facility staff interviews, debriefs, and surveys.

### 2.7. Data Analysis

The service delivery bundle will be reviewed as a prospective, observational program assessment using a pre–post design.

- Quantitative Analysis: Clinical and implementation indicators (Table 2 and Table 3) from the baseline period (3 months prior to implementation) will be compared with the final period (last 3 months of the project). Data will be analyzed using analysis of variance, unpaired and paired t-tests, and chi-square analysis as appropriate.
- Qualitative Analysis: Qualitative data from staff interviews and debriefs will be transcribed and subjected to thematic analysis to identify operational bottlenecks and assess the acceptability and feasibility of the service delivery bundle.

### 2.8. Ethical Considerations

Because this protocol is structured as a service delivery program review rather than a clinical trial, it does not involve human subjects research. All clinical elements of the newborn and maternal packages are standard, evidence-based practices with ample prior evidence of benefits and minimal risk of harm. The assessment relies exclusively on the secondary analysis of routine programmatic data and information mandated by the Ministry of Health and Sanitation (MoHS). All extracted clinical data will be fully anonymized and aggregated at the facility level, ensuring the de-identification of patient outcomes. Prior to implementation, the project was carefully evaluated by the MoHS and approved.

## 3. Expected Results and Discussion

### 3.1. Scientific and Public Health Significance

Sierra Leone continues to bear a disproportionate share of the global burden of maternal and neonatal mortality. Over a period of three years, we anticipate that this bundle will address the “second delay”—the delay in identifying and treating complications—by equipping facilities with the capacity to recognize and treat respiratory distress and hemorrhage immediately. By validating a bundle that includes both the Vayu bCPAP system and POCUS, we anticipate that this review will contribute to the global evidence base for low-cost, non-invasive respiratory support and maternal risk reduction in LMICs.

### 3.2. The Innovations

Two key innovations of this bundle are the expansion of bCPAP therapy and maternity unit point-of-care ultrasound coverage. Effective oxygen delivery requires blending and CPAP to avoid hyperoxic injury, a capacity which is often lacking in resource-constrained hospitals. Furthermore, the introduction of handheld ultrasound (Butterfly iQ3) into the delivery ward represents a task-shifting approach that we anticipate will empower midwives to perform advanced risk assessments at the bedside.

This bundle contains key clinical tools and practices with demonstrated effectiveness in other low-and-middle-income (LMIC) contexts. The Safer Birth Bundle of Care program in Tanzania achieved significant reductions in perinatal mortality through the deployment of similar scaling, service delivery improvements, and sustainability approaches using similar clinical tools. In a separate study from Tanzania, Vayu bCPAP implementation was associated with immediate improvements in respiratory function.

### 3.3. Comprehensive “Bundle” Approach

In contrast to single-intervention clinical studies, this protocol evaluates the implementation of a comprehensive service delivery bundle. We posit that improving newborn survival is impossible without simultaneously addressing maternal complications. By combining the WHO ENCC with maternal risk assessment and the WHO PPH Package, the implementation addresses the dyad of mother and baby, ensuring that survival for one is not compromised by the death of the other.

### 3.4. Supportive Supervision and Checklists

Evidence suggests that classroom training alone is insufficient to change clinical practice. Supportive supervision, defined as a collaborative, mentorship-driven process, rather than traditional inspection, has been shown to significantly improve health worker performance and motivation in low-resource settings [28,29,30]. Furthermore, the implementation of delivery observation using structured checklists and tools has been shown to increase adherence to essential birth practices and reduce severe complications [31]. By combining LDHF practice with ongoing supervisory checklists, we anticipate that this study will address the “know–do” gap often seen in maternal health programs.

### 3.5. Limitations

The protocol assessment will involve a pre–post design without a control group; therefore, the findings may be influenced by concurrent government programs, hospital maintenance issues, or external factors. Additionally, the reliability of the retrospective baseline data depends on the quality of the historical facility registers; however, this factor will be mitigated by a robust data cleaning and cross-verification process. The contribution of supplies and consumables, both to support the innovations and in addition to IPC and routine supplies, cannot be independently measured.

### 3.6. Sustainability and Policy Implications

Sustainability was incorporated into the protocol through an 18-month “Sustainability Phase” aimed at transitioning ownership to the MoHS. By utilizing a “train-the-trainer” model and integrating national trainers, the project will ensure that capacity will remain within the system after the implementation phase concludes.

## 4. Conclusions

This bundle of service delivery improvements represents a significant effort to operationalize national child and maternal survival priorities within a network of nine facilities. By combining high-impact training and technologies with a sustainable mentorship model, the aim of this bundle is to bridge the gap between policy intent and clinical reality, improving quality of care and reducing the burden of preventable deaths.

## Figures and Tables

**Table 1:** Baseline characteristics and target training populations of participating healthcare facilities (2023).

Facility Name	Location (City)	Number of Annual Births	Maternal Deaths (2023)	Neonatal Deaths (2023)	Number of Annual C/S	Healthcare Workers Trained in Past 2 Years	Total Healthcare Workers to Be Trained
Bo Government Hospital	Bo	3226	34	191	1053	28	23
Jenner Wright Clinic	Freetown	412	17	6	14	13	-
King Harman Road Government Hospital	Freetown	712	8	15	331	9	87
Kissy CHC	Freetown	440	17	4	3	19	-
Princess Christian Maternity Hospital	Freetown	7099	63	464	3016	12	125
Rokupa Government Hospital	Freetown	1495	1	54	599	5	65
Ross Road CHC	Freetown	626	3	1	3	29	-
St. Anthony’s CHC	Freetown	511	19	2	5	27	-
Waterloo CHC	Waterloo	1015	17	11	20	8	23

**Table 2:** Education and training.

Category	Indicator	Definition
Training uptake	Number of healthcare workers trained	Total number of midwives, nurses, physicians, and other staff completing WHO ENCC and PPH training modules
	Training completion against targets	Number completing training/facility training target
Skills retention	Frequency of LDHF practice sessions	Number of documented low-dose, high-frequency practice sessions per facility
	OSCE performance scores	Percentage of participants achieving competency on Objective Structured Clinical Examinations (OSCEs)
	Skills retention at 12 months	Percentage of trained staff maintaining competency at 12-month reassessment
Mentorship and supervision	Mentorship visits completed	Number of supportive supervision and mentorship visits conducted per facility
	Practice corner utilization	Frequency of use of facility-based LDHF practice stations

**Table 3:** Maternal and newborn measures with definitions.

Category	Indicator	Definition
Maternal indicators	PPH incidence (500 cc)	[# of PPH incidence (500 cc)]/[total births]
	Uterotonics used for PPH prevention	[# of uterotonics used for PPH prevention]/[total births]
	Direct obstetric case fatalities	[# maternal deaths]/[# of total births]
	Admission triage scans	[# of ultrasound scans]/[# of maternal admissions]
	Abnormal scan findings	[# of abnormal scans]/[# of scans]
Neonatal indicators	Fresh stillbirths	[# of fresh stillbirths]/[total births]
	Neonatal case fatality rate < 24 h	[# of neonatal deaths < 24 h]/[total births]
	Neonatal case fatality rate < 48 h	[# of neonatal deaths < 48 h]/[total births]
	Neonatal transfers from maternity	[# of neonatal transfers from maternity]/[total births]
	Sick newborns on bCPAP	[# on bCPAP]/[# of SCBU admissions]
	Survival of sick newborns	[# survive SCBU admission]/[# of admissions]

# = number.

## Data Availability

Data sharing is not applicable to this article as no new data were created or analyzed in this protocol.

## References

[1] United Nations Inter-Agency Group for Child Mortality Estimation (UN IGME) (2024). Levels and Trends in Child Mortality: Report 2024.

[2] World Health Organization (WHO) (2025). Trends in Maternal Mortality 2000 to 2023: Estimates by WHO, UNICEF, UNFPA, World Bank Group, and UNDESA/Population Division.

[3] Statistics Sierra Leone (Stats SL), ICF (2020). Sierra Leone Demographic and Health Survey 2019.

[4] United Nations Children’s Fund (UNICEF) (2023). Country Office Annual Report 2023: Sierra Leone.

[5] Versantvoort J.M., Kleinhout M.Y., Ockhuijsen H.D., Bloemenkamp K., de Vries W.B., van Den Hoogen A. (2020). Helping Babies Breathe and its effects on intrapartum-related stillbirths and neonatal mortality in low-resource settings: A systematic review. Arch. Dis. Child..

[6] Dol J., Campbell-Yeo M., Murphy G.T., Aston M., McMillan D., Richardson B. (2018). The impact of the Helping Babies Survive program on neonatal outcomes and health provider skills: A systematic review. JBI Database Syst. Rev. Implement. Rep..

[7] Clark R.B., Dhungana R., Chalise M., Visick M.K. (2023). Scale-up of Neonatal Resuscitation Training and Skill Retention in Five Provinces of Nepal. Asia Pac. J. Public Health.

[8] Wall S.N., Lee A.C.C., Niermeyer S., English M., Keenan W.J., Carlo W., Bhutta Z.A., Bang A., Narayanan I., Ariawan I. (2009). Neonatal resuscitation in low-resource settings: What, who, and how to overcome challenges to scale-up?. Int. J. Gynaecol. Obstet..

[9] Emmanuel A., Kain V.J., Forster E. (2019). The Impact of the World Health Organization Essential Newborn Package on Newborn Care Practices and Survival Rates in Sub-Saharan Africa: A Systematic Literature Review. Int. J. Childbirth.

[10] Kamala B.A., Ersdal H.L., Moshiro R.D., Guga G., Dalen I., Kvaløy J.T., Bundala F.A., Makuwani A., Kapologwe N.A., Mfaume R.S. (2025). Outcomes of a Program to Reduce Birth-Related Mortality in Tanzania. N. Engl. J. Med..

[11] Tayler A., Ashworth H., Bou Saba G., Wadhwa H., Dundek M., Ng E., Opondo K., Mkony M., Moshiro R., Burke T. (2022). Feasibility of a novel ultra-low-cost bubble CPAP (bCPAP) System for neonatal respiratory support at Muhimbili National Hospital, Tanzania. PLoS ONE.

[12] Ahn E., Perlman J., Mselle M., Sechu A., Perlman J. (2025). Implementation of a novel bubble continuous positive airway pressure system. J. Perinatol..

[13] Banik G., Halim M.A., Abdullah A.S.M., Oishee I., Boyce C., Dey S.K., Shabuj M.K.H. (2024). Vayu bubble continuous positive airway pressure is a promising solution with favorable treatment outcomes for respiratory distress syndrome in newborns: A qualitative study in Bangladesh. Front. Pediatr..

[14] Rauschendorf P., Saba G.B., Meara G.K., Roodaki N., Conde-Agudelo A., Garcia D.E.C., Burke T.F. (2023). Effectiveness of a novel bubble CPAP system for neonatal respiratory support at a referral hospital in the Philippines. Front. Pediatr..

[15] Ng E., Dundek M., Burke T.F. (2022). Evaluation of an innovative low-flow oxygen blender system. Front. Pediatr..

[16] Deuber C., Terhaar M. (2011). Hyperoxia in Very Preterm Infants. J. Perinat. Neonatal Nurs..

[17] Shafiq Y., Caviglia M., Bah Z.J., Tognon F., Orsi M., Kamara A.K., Claudia C., Moses F., Manenti F., Barone-Adesi F. (2024). Causes of maternal deaths in Sierra Leone. BMJ Open.

[18] World Health Organization (WHO) (2023). WHO Recommendations on the Assessment of Postpartum Blood Loss and Use of a Treatment Bundle for Postpartum Hemorrhage.

[19] Gallos I., Devall A., Martin J., Middleton L., Beeson L., Galadanci H., Al-Beity F.A., Qureshi Z., Hofmeyr G.J., Moran N. (2023). Randomized trial of early detection and treatment of postpartum hemorrhage. N. Engl. J. Med..

[20] Akter S., Forbes G., Corona M.V., Miller S., Althabe F., Coomarasamy A., Gallos I.D., Oladapo O.T., Vogel J.P., Lorencatto F. (2023). Perceptions and experiences of the prevention, detection, and management of postpartum hemorrhage: A qualitative evidence synthesis. Cochrane Database Syst. Rev..

[21] Gelaw K.A., Atalay Y.A., Azeze G.A., Yitayew A.M., Gebeyehu N.A. (2024). Knowledge and factors associated with active management of the third stage of labor in sub-Saharan Africa: A systematic review and meta-analysis. Int. J. Gynaecol. Obstet..

[22] Gelaw K.A., Assefa Y., Birhan B., Gebeyehu N.A. (2023). Practices and factors associated with active management of the third stage of labor in East Africa: A systematic review and meta-analysis. BMC Pregnancy Childbirth.

[23] Kim E.T., Singh K., Moran A., Armbruster D., Kozuki N. (2018). Obstetric ultrasound use in low- and middle-income countries: A narrative review. Reprod. Health.

[24] Ministry of Health and Sanitation (MoHS) (2021). National Health Sector Strategic Plan 2021–2025.

[25] Ministry of Health and Sanitation (MoHS) (2023). Sierra Leone Child Survival Action Plan 2023–2025.

[26] World Health Organization (WHO) (2024). Essential Newborn Care Course.

[27] American Academy of Pediatrics (AAP) (2021). Helping Babies Breathe.

[28] Hill Z., Dumbaugh M., Benton L., Källander K., Strachan D., Asbroek A.T., Tibenderana J., Kirkwood B., Meek S. (2014). Supervising community health workers in low-income countries: A review of impact and implementation issues. Glob. Health Action.

[29] Avoka C., Awoonor-Williams J.K., Adongo P.B. (2017). Effect of support supervision on maternal and newborn health services and practices in Rural Eastern Uganda. Reprod. Health.

[30] Fernández-Elorriaga M., Fifield J., Semrau K.E.A., Lipsitz S., Tuller D.E., Mita C., Cho C., Scott H., Taha A., Dhingra-Kumar N. (2025). Impact of the WHO Safe Childbirth Checklist on birth attendant behavior and maternal-newborn outcomes: A systematic review and meta-analysis. Int. J. Gynaecol. Obstet..

[31] Spector J.M., Agrawal P., Kodkany B., Lipsitz S., Lashoher A., Dziekan G., Bahl R., Merialdi M., Mathai M., Lemer C. (2012). Improving quality of care for maternal and newborn health: Prospective pilot study of the WHO safe childbirth checklist program. PLoS ONE.

